# Evaluation of Dietary Intakes and Nutritional Knowledge in Thai Patients with Type 2 Diabetes Mellitus

**DOI:** 10.1155/2018/9152910

**Published:** 2018-12-20

**Authors:** Yotsapon Thewjitcharoen, Phawinpon Chotwanvirat, Annapann Jantawan, Nantaporn Siwasaranond, Sunee Saetung, Hataikarn Nimitphong, Thep Himathongkam, Sirimon Reutrakul

**Affiliations:** ^1^Diabetes and Thyroid Center, Theptarin Hospital, Bangkok, Thailand; ^2^Division of Endocrinology and Metabolism, Department of Medicine, Faculty of Medicine Ramathibodi Hospital, Bangkok, Thailand; ^3^Division of Endocrinology, Diabetes and Metabolism, Department of Medicine, University of Illinois at Chicago, Chicago, Illinois, USA

## Abstract

**Introduction:**

Most nutritional guidelines for diabetes management emphasize the importance of having individualized goals, away from a one-size-fits-all approach. However, there is a dearth of information on the dietary intakes and nutritional knowledge of Thai patients with type 2 diabetes mellitus (T2DM). This study is aimed at clarifying dietary intakes in relationship to glycemic control and at examining nutritional knowledge among Thai patients with T2DM.

**Materials and Methods:**

A cross-sectional study of outpatients with T2DM at Theptarin Hospital and Ramathibodi Hospital (Bangkok, Thailand) was performed to assess dietary intakes by food records. Diabetes nutritional knowledge and dietary self-care behavior was also evaluated.

**Results:**

A total of 304 Thai patients with T2DM (female 52.6%, mean age 57.4 ± 10.9 years, body mass index (BMI) 27.3 ± 4.8 kg/m^2^, and baseline A1C 7.2 ± 1.3%) participated in the study. The mean daily calorie intake was 1427 ± 425 kcal, and mean intake for each macronutrient was acceptable (carbohydrate 52%, protein 17%, and fat 31%). However, the intake of free sugar was much higher (12.1 ± 5.8% of total daily energy intake) and dietary fiber intake (9 grams per day) was much lower than recommended. There were no correlations between dietary intake and glycemic control. A subset of patients (*N* = 213) completed the diabetes nutritional knowledge survey. There was no association between diabetes nutritional knowledge and the actual dietary self-care behavior.

**Conclusion:**

These results indicate that compliance of Thai patients with T2DM to dietary recommendations is not completely satisfactory, especially for free sugar and dietary fiber intakes. Addressing the reality of how patients with T2DM eat in their daily lives and their knowledge gaps would enable them to adhere to medical nutrition therapy.

## 1. Introduction

Despite the evidence and recommendations in medical nutritional therapy (MNT) from various guidelines for diabetes management [1–3], the importance of individual nutritional needs based on personal and cultural preferences is still paramount in patient counseling. Patients with diabetes who are able to adhere to dietary self-care recommendations often have a better glycemic control, leading to fewer diabetic complications [4]. However, motivating patients to achieve dietary self-care behaviors is challenging and needs ongoing efforts between patients and multidisciplinary teams. Since 2014, the American Diabetes Association (ADA) has been emphasizing the need for individualized nutrition therapy. Therefore, nutrition assessment is an important step to identify problems in order to make appropriate recommendations according to each individual's needs.

Consumption patterns around the world have become converging toward a Western diet, characterized by more sugar-sweetened beverages, highly processed foods and animal-based foods, and fewer fruits and vegetables [5, 6]. Thailand has been undergoing major industrial and social transformations with rapid economic growth and development for over half a century. In 2011, after more than 20 years as a lower-middle-income country, the World Bank upgraded Thailand to upper-middle-income status [7]. The availability, affordability, and popularity of wide assortment of foods and tropical fruits, most of them high in natural sugars, in Thailand is an enormous obstacle to dietary adherence in people with diabetes [8–10]. Despite the known importance of diet modification, many people with diabetes struggle to adopt and maintain a clinically recommended diet.

In Thailand, there have been no epidemiological studies on dietary intakes in patients with diabetes at the national level. Nutrition assessment of patients with diabetes is challenging, as conventional food recalls are vulnerable to underreporting especially in obese individuals [11–13]. One of the most frequently used methods of determining habitual dietary intake is the food frequency questionnaire (FFQ). Because the FFQ is simple to implement in large-scale studies, it has been widely used to evaluate associations between dietary intakes and outcomes of interest [14]. However, while the FFQ is a useful dietary assessment tool for the general healthy populations, it does not address the cultural variability and diet-related conditions. Currently, a diabetes-specific FFQ in the context of Thai food and culture is not available. In addition, there has been very limited data on the dietary intakes and nutritional knowledge of Thai patients with type 2 diabetes mellitus (T2DM).

**Figure 1:**
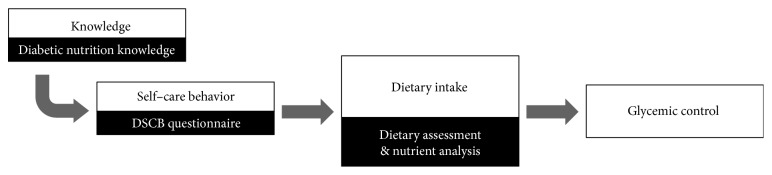
Objective diagram of this study.

The primary objective of this study was to characterize dietary intakes of patients with T2DM in Thailand. The secondary objectives were (1) to assess the relationship between dietary intake and glycemic control, (2) to assess the relationship between diabetes nutritional knowledge and actual dietary self-care behavior in order to identify knowledge gaps in adhering to medical nutrition therapy, and (3) to examine the relationship between the actual dietary self-care behavior and dietary intakes. These objectives are illustrated in Figure 1.

## 2. Materials and Methods

### 2.1. Research Settings and Participants

This is a cross-sectional study of Thai adults diagnosed with T2DM seen at out-patient diabetes clinics at Theptarin Hospital and Ramathibodi Hospital (both located in Bangkok metropolitan area). For Theptarin Hospital, a total of 213 patients were recruited between 2015 and 2017. Inclusion criteria were (1) ages between 25 and 85 years and (2) diagnosis of T2DM and willingness to participate in the study with the ability to comprehend relevant information. Patients with life-threatening illnesses, impaired renal function (estimated glomerular filtration rate less than 60 mL/min/1.73 m^2^), pregnant women, participants with recent weight loss or weight gain of more than 5% of current body weight within 3 months, and those who had type 1 diabetes mellitus or other chronic conditions that may influence physical activity such as stroke and cancer were excluded.

For Ramathibodi Hospital, we used secondary data from a study evaluating sleep characteristics in patients with T2DM from 2014–2015 [15]. This study recruited adults with T2DM who were being followed in the endocrinology clinic at the Faculty of Medicine Ramathibodi Hospital, Mahidol University. Exclusion criteria were (1) having been previously diagnosed with obstructive sleep apnea, (2) being pregnant or performing shift work, and (3) patients with a history of congestive heart failure or low ejection fraction, chronic obstructive pulmonary disease, end-stage renal disease or severe chronic liver disease such as cirrhosis, stroke, permanent pacemaker placement, and use of certain medications (opioids/narcotics, alpha adrenergic blockers, clonidine, methyldopa, and nitroglycerin). A total of 91 patients with complete dietary intakes data were recruited from 2014 to 2015.

Patients who did not complete food records and whose total daily energy intake was less than 500 kcal or greater than 5000 kcal (*n* = 11) were excluded. These exclusions were established based on unrealistic dietary intakes of the general population in nutritional research [14]. All patients provided written informed consent, and the study protocol was approved by the Theptarin Hospital ethics committee (EC no. 07/2016) and by Ramathibodi Hospital, Mahidol University (IRB no. 02-57-22).

### 2.2. Demographic and Clinical Data

Eligible patients were interviewed for information on sociodemographic parameters which include age, gender, marital status, educational level, place of residence, smoking, and alcohol consumption. Duration of diabetes, current diabetic treatments, and laboratory data (hemoglobin A_1c_ (A1C), lipid profiles, and serum creatinine) were obtained from medical records. Only values recorded for the past three months were used for the study. Body mass index (BMI) was calculated from weight and height information.

### 2.3. Dietary Assessment

At Theptarin Hospital, the dietary assessment was determined by a 3-day food record (two weekdays and one weekend day). Patients were given oral instructions by trained dietitians on how to record their food intakes and were shown how to use a household scale. Written instructions were incorporated in the food diaries, along with contact information. Information on the type, brand names, and amount of food consumed was collected. For verification and estimation of the size of individual food portions, some patients were instructed to send digital food photography through mobile phone to increase the accuracy of portion size estimation. The importance of maintaining regular diets and recording all foods and drinks consumed during the study was emphasized. The quality control of all food diaries was handled and reviewed by an experienced research dietitian to avoid inconsistency and to maintain accurate data entries.

At Ramathibodi Hospital, dietary assessments were done by a 7-day food record. Oral instructions were given by a nurse with extensive experience in nutritional evaluation. Measuring cups and spoons were given to help the patients assess their food portions more accurately. The completeness of the record was reviewed upon returning of the food log by the same nurse, with emphasis on types, cooking methods, and portions of food consumed.

The food codes were those used in the Thai Dietary Database (fourth edition), Institute of Nutrition, Mahidol University [16]. The calculation of nutrients was done by the INMUCAL-Nutrients software version 3 [17]. Incomplete food records and unrealistic values of reporting energy intakes/nutrients were excluded from the analysis.

### 2.4. Diabetes Nutritional Knowledge

The assessment of patients' diabetes nutritional knowledge was done by the validated “Theptarin DM questionnaire” [18] in patients recruited by Theptarin Hospital (*N* = 213). The questionnaire consists of 10 questions from 4 domains, which included diabetes-specific food choices, basic carbohydrate counting, general diabetes knowledge, and diabetes treatment knowledge. After three experts validated the content of each question, validation analysis was performed according to the standard psychometric evaluation (internal validity, construct validity, and test-retest reliability) in two populations (diabetes care professionals and non-DM people). Finally, the questionnaire was administered in Thai patients with T2DM. Reliability test using Cronbach's alpha coefficient yielded 0.692 which is considered reliable for a newly developed instrument. Only domains of diabetes-specific food choices and basic carbohydrate counting were used in this current analysis (total score = 5). The scores were classified into three categories: low (score less than 3), moderate (score 3-4), and high (score = 5). The details of adapted “Theptarin DM questionnaire” can be found in Appendix 1.

### 2.5. Dietary Self-Care Behavior (DSCB) Questionnaire

Dietary self-care behavior (DSCB) was evaluated in patients from Theptarin Hospital (*N* = 213) by a questionnaire modified (with permission) from a previously published tool by Taiwanese researchers [19]. The questionnaire assesses patients' reports of the self-care recommendations they had received from healthcare providers and adherence to seven reported diet-related self-care behaviors. The details of DSCB included the following 7 items: (1) adhering to a diabetic meal plan, (2) having meals every day at the same time and with the same amount of carbohydrate, (3) following the diabetes food exchange system, (4) counting carbohydrates, (5) reducing dietary fat, (6) consuming foods high in fiber, and (7) keeping a daily food record. Items questioning each of these behaviors were constructed using a 5-point Likert scale to rank adherences. The frequency of diabetes self-care behavior was categorized into low adherence (never and seldom) and high adherence (often and always). The details of DSCB can be found in Appendix 2.

## 3. Statistical Analysis

Continuous variables were presented as mean (±standard deviation, SD) or median (interquartile range), and categorical variables were presented as proportions. Comparisons between two groups were done using unpaired Student's *t*-test for continuous data. To explore the association between diabetes nutritional knowledge and diabetes self-care behaviors, comparisons of knowledge scores between those with high vs. low adherence to self-care behavior were performed. Dietary intakes between patients with high adherence and those with low adherence to diabetes self-care behavior and those with good vs. poor glycemic control (A1C < 7.0% vs. ≥7.0%) were also compared. Multiple regression analyses by stepwise regression technique were performed as a post hoc analysis to define the independence factors on glycemic control. The dependent variable was A1C (as continuous data) and independent variables included age (as continuous data), sex (as categorical data), BMI (as continuous data), duration of DM (as continuous data), insulin usage (as categorical data), daily energy intakes (as continuous data), and macronutrient intakes (as continuous data). All statistical analyses were conducted using the Statistical Package for the Social Sciences (version 22.0; SPSS, Chicago, IL, USA). *p* value ≤ 0.05 was considered statistically significant.

## 4. Results

### 4.1. Baseline Characteristics

A total of 304 patients (females 52.6%, mean age 57.4 ± 10.9 years, BMI 27.3 ± 4.8 kg/m^2^, median duration of diabetes 14 years, baseline A1C 7.2 ± 1.3%, and insulin treatment 21%) participated in the study. Most of the patients were married (74.2%) and live in urban areas (74.8%). More than 50% of recruited patients had good glycemic control based on having A1C < 7.0%. The majority of patients were overweight or obese (80.2%) based on the WHO-recommended BMI cut-offs for Asians [20]. Baseline characteristics of patients are shown in Appendix 3. Only 14 patients (5%) were on diet control alone, and this group of patients had a shorter duration of diabetes when compared with those who were on antidiabetic medications (median duration of DM 4 years vs. 10 years in the latter group). When comparing the patients between sites, those from Ramathibodi Hospital were slightly older and had worse metabolic profiles (more obese, higher rates of hypertension and dyslipidemia). However, there were no differences in duration of diabetes and education levels between the patients from the two sites. Insulin usage rate was higher in Ramathibodi Hopsital when compared with Theptarin Hospital (30% vs. 12%, *p* value 0.012).

### 4.2. Daily Energy Intakes and Nutrient Intakes

The mean caloric intake was 1427 ± 425 kcal/day, and mean intake for each macronutrient was acceptable (carbohydrate 52%, protein 17%, and fat 31%). As shown in Table 1, the mean carbohydrate intake was 197 ± 57 grams/day for men and 173 ± 51 grams/day for women, representing 51% and 52% of their total energy intake, respectively. The intakes of saturated fat and free sugar were much higher and dietary fiber intake was much lower than recommended (saturated fat ≤ 7%, free sugar ≤ 5%, and dietary fiber intake ≥ 14 grams per 1000 kcal). Only 32.7%, 11.8%, and 1.6% of patients met recommendations for saturated fat (<10% of total energy), free sugar (≤5% of total energy), and fiber intake (≥14 grams per 1000 kcal), respectively. Notably, the mean intake of estimated dietary calcium was only 366 ± 164 mg/day and only 0.5% of patients consumed at least 1000 mg/day from dietary sources.

When compared nutrient intakes between both sites, there were higher daily energy intakes, protein intakes, fat intakes, sodium intakes from Theptarin patients. However, the excessive consumption of free sugar and inadequate intake of dietary fiber were similar in patients from both sites.

### 4.3. The Relationship between Dietary Intake and Glycemic Control

The demographic data between patients who achieved good glycemic control and those who did not achieve glycemic control are shown in Appendix 4. Only patterns of DM treatments showed the statistically significant between 2 groups. As shown in Table 2, there was no association between total energy and macronutrient intake between patients who achieved good glycemic control (A1C < 7.0%) and patients who did not (A1C ≥ 7.0%). In a subgroup of patients who were on diet control alone and achieved good glycemic control (A1C < 7.0%), there were no differences in dietary intake data when compared with those who were on antidiabetic medications and achieved good glycemic control (A1C < 7.0%) (data not shown). In the subgroup analysis of patients from each site, there was also no association between total energy and macronutrient intake between those who achieved good glycemic control (A1C < 7.0%) and those who did not (A1C ≥ 7.0%) as shown in Table 3.

### 4.4. The Relationship between Diabetes Nutritional Knowledge, Dietary Self-Care Behavior, and Macronutrient Intake

**Figure 2:**
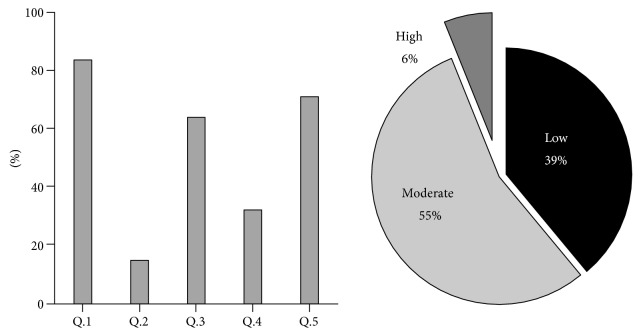
Results of individuals' knowledge of diabetes and diabetes nutritional knowledge by the validated “Theptarin DM questionnaire” (*N* = 213 cases).

The subset of patients (Theptarin Hospital, *N* = 213) completed diabetes nutritional knowledge with adapted “Theptarin DM questionnaire” and dietary self-care behavior (DSCB) questionnaire. As shown in Figure 2, the mean diabetic knowledge score was 2.7 ± 1.2 out of a total of 5 and only 6% of patients obtained high scores. Based on the DSCB questionnaire (Table 4), 70% of the patients had received previous dietary advice from certified dietitians but less than half applied the knowledge to daily eating behaviors. Only 12% of the patients kept food diary to monitor their food intakes.

As shown in Table 5, there was no difference in diabetes nutritional knowledge scores between patients with low and high adherence to dietary self-care behavior, implying that routine dietary practices did not depend on the level of knowledge. The relationship between the actual dietary self-care behavior and dietary intake revealed that patients with high adherence in following dietary exchange method tended to consume more protein and dietary fiber compared with those with low adherence (*p* values 0.030 and 0.047, respectively). However, there was no difference between total energy and carbohydrate intakes between patients who had high vs. low adherence to diabetes self-care behavior (Table 6).

### 4.5. Adjusted Daily Energy Intakes and Nutrient Intakes after Exclusion of Underreporters

As underreporting is a well-recognized phenomenon in collecting dietary data, we further analyzed the data by excluding underreporters using previously published methods as shown in Appendix 5. After the exclusion of underreporters, percentages of macronutrient intakes remained unchanged, and excessive consumption of free sugar and inadequate intake of dietary fiber were consistently observed.

### 4.6. Multiple Regression Model Explaining Glycemic Control

In multiple regression analyses by stepwise regression technique, only insulin usage and longer duration of diabetes were found to be significantly associated with poorer glycemic control (*p* value < 0.05) as shown in Table 7. Daily energy and nutrients data were not found to be associated with A1C.

## 5. Discussion

The current study revealed that Thai patients with diabetes consume excessive amount of saturated fat and free sugar, but very low dietary fiber. Even though the average carbohydrate intake was acceptable at 52% of total energy intake, patients in our study consumed more than 3 times the recommended amount of free sugar. While the majority of patients reported receiving previous nutritional counseling as revealed by a subset of patients who completed DSCB, less than half practiced what they have learned. Moreover, there were no associations between the knowledge and behavioral adherence in general. This indicated additional barriers in knowledge application, which may include the techniques utilized in teaching and motivating the patients, patients' perception, and cultural and socioeconomic factors. These results suggest that better strategies are needed to help these patients with T2DM achieve their dietary goals and thereby better control their diabetes. Even though our study did not find association between dietary intakes and achievement of glycemic control, those who could achieve glycemic control demonstrated higher rate of being on diet control alone as their diabetic treatment. This observation could be interpreted that diet interventions still play a vital role in management of patients with early stage of T2DM (i.e., those with a short duration of diabetes). Based on the results of multiple regression analysis, patients who were not on insulin treatment and patients with short duration of diabetes might be the most likely candidates who could achieve glycemic control with only MNT and lifestyle intervention. In the future study, it should be examined whether diet quality would be more important than specific nutrients and nutrient levels in Thai patients with T2DM.

Carbohydrate is a key macronutrient that influences postprandial glucose levels. Recommendations of carbohydrate intake for patients with diabetes have gone through several revisions. The 2004 position statement from the ADA recommended the total carbohydrate intake not to exceed 65% of total calories/day but not less than 130 grams per day [21]. The latest recommendations are from 2014, in which the ADA changed their position regarding carbohydrate intake to conclude that there is no definite evidence of an ideal amount of carbohydrate intake for people with diabetes [1, 22–25]. Our study found that carbohydrate intake was at 52% which was an acceptable level when considered by ADA statement in 2004. In Thailand, local guidelines were also developed based on the ADA recommendations in 2014, with a focus on carbohydrate counting and food exchange to control postprandial blood glucose [26]. The summary of our data compared with the current Thai guideline and ADA recommendations is demonstrated in Table 8. Rice is the main source of carbohydrate in a typical Thai diet, but sweet tropical fruits (such as orange, ripe mango, pineapple, and rambutan) are also popular depending on the season. The availability of nonsweet fruits is very limited in Thailand; therefore, some patients with diabetes tend to have worse glycemic control during certain seasons due to the availability of high-glycemic index seasonal fruits as shown in a recent qualitative study from Sri Lankan people with T2DM [27]. This study found that patients who practiced dietary exchange method consumed more protein and dietary fiber than those who were not using this method. Our findings confirmed the utility of the food exchange list as an appropriate tool to select healthier food choices.

Our patients consumed an average of 43 grams of free sugar or 12% of their total calories. Overconsumption of sugar is a major contributor to obesity and heart disease in people with T2DM [28, 29]. Free sugar from soft drinks, fruit drinks, baked goods, and processed foods, of which patients might not be aware, is a severely underrecognized problem in our patients. Another interesting finding from our results was a very low intake of dietary fiber. The mean daily intake of fiber was only 9 grams, and only 1.6% of patients consumed adequate daily fiber according to recommendation. Most dietary guidelines recommend consumption of two servings of fruits and three servings of vegetables daily. The ADA suggested that patients with T2DM should consume at least 14 grams of fiber for every 1000 calories consumed, which translates into a daily intake of approximately 25 grams for women and 38 grams for men [1]. Moreover, a recent meta-analysis suggested that higher dietary fiber intake was associated with reduced risk of all-cause mortality [30]. Local traditional Thai dishes from different regions contain a lot of vegetables and herbs. However, urban lifestyles as in Bangkok, together with various factors that influence food choices, led to insufficient fiber intake. Policies that enhance the affordability of nonsweet fruits and vegetables are needed to meet these recommendations. A session with a registered dietitian or other qualified healthcare professional can facilitate meal planning to increase dietary fiber intake.

In many low-income and middle-income countries, undernutrition has been a major concern. However, the diet-related burden of disease in these regions is shifting towards noncommunicable diseases and less than 50% of treated patients with diabetes could achieve levels of A1C < 7.0% [31]. In management of a chronic condition, knowledge is a prerequisite to patient empowerment [32]. Nonetheless, we demonstrated here that dietary self-care behavioral adherence did not correlate with knowledge, suggesting that other factors play a role in applying the knowledge. Medical nutrition therapy is an integral part of diabetes self-management and considered an art as well as science since the influence of sociocultural and religious backgrounds needs to be considered when giving guidance for realistic food choices based on patients' own usual eating patterns [33]. Therefore, dietary behaviors and objective data from nutritional assessments need to be investigated in order to provide individualized meal plans and promote healthy eating behaviors. Additionally, educational styles should shift from one-way communication or lecture-based to interactive, personalized, and realistic behavioral goal setting by various educational methods such as motivational interviewing, along with follow-ups and coaching as needed to ensure adherence and desired outcomes. These will help patients with T2DM develop realistic and sustainable strategies [34, 35].

Several limitations of this study should be noted. First, although more than 300 patients participated in this cross-sectional study, the patients were recruited from only 2 hospitals in the downtown area of Bangkok. The generalizability of the results should be carefully considered because commonly consumed foods and food availability differ between areas and countries. Second, nutrition assessments in this study were done with 3-7-day food records and supplemented with image-assisted assessment of dietary intake in some cases (over 60% of participants). This may not capture usual dietary intakes and could be one of the reasons that we did not find a relationship between dietary intake and glycemic control. We acknowledged the universal problem of underreporting estimated energy intakes in this study and tried to determine the proportion of underreporters using previously published methods in estimating resting energy expenditure (REE) as summarized in Appendix 5. We found that our current data might underestimate at least 18-35% in daily energy intakes, but the distribution of macronutrient intakes remained unchanged after excluding underreporters. Even though the quality of dietary intake data from quantification of portion sizes was more reliable when compared with food recalls or FFQ [36, 37], they were labor-intensive procedures and still presented underreporting. Patients may have wanted to appear highly health conscious, due to social desirability bias. This effect could not be excluded and might have caused the nonsignificant relationship between nutritional knowledge and glycemic control in our patients. Because short-term recall and diet recording methods are underrepresentative of usual dietary intakes and did not reflect seasonal variation in food intake, a validated diabetes-specific FFQ in Thai patients with T2DM should be developed as a dietary assessment tool in epidemiological studies. The relative ease of administration/data analysis, its consideration of seasonal intake variation, and inexpensiveness make FFQ a preferred choice for nationwide nutrition surveys [38]. In addition, focusing on food based healthy eating patterns rather than any single nutrient-based assessment may better aid in predicting outcomes in patients with diabetes. Finally, certain food groups in the actual intake from patients were not available in the INMUCAL-Nutrients software, so their intakes had to be estimated from similar food items.

In conclusion, our study indicated that compliance of Thai patients with T2DM to dietary recommendations is not completely satisfactory. In particular, Thai patients have high intakes of saturated fat and free sugars while having insufficient intake of dietary fiber. In addition, nutritional knowledge was not a predictor of dietary intakes. Strategies in enhancing dietary compliance and a development of a more practical diabetes-specific nutritional assessment tool to be used in wider nutritional surveys in the context of Thailand should be considered.

## Figures and Tables

**Table 1:** Daily energy and nutrient intakes (*N* = 304 cases) in all patients and divided by site.

	Total (*n* = 304)	Total (*n* = 304)		*p* value	Total (*n* = 304)		*p* value
		Men (144)	Women (160)		Ramathibodi (91)	Theptarin (213)	
Energy (kcal)	1427 ± 425	1556 ± 422	1312 ± 395	<0.001	1272 ± 346	1494 ± 439	<0.001
Carbohydrate (g)	185 ± 55	197 ± 57	173 ± 51	<0.001	177 ± 53	188 ± 56	0.121
Protein (g)	60 ± 21	66 ± 20	55 ± 20	<0.001	51 ± 15	64 ± 22	<0.001
Fat (g)	49 ± 20	54 ± 20	44 ± 19	<0.001	40 ± 15	53 ± 21	<0.001
Cholesterol (mg)	270 ± 133	298 ± 134	245 ± 129	<0.001	234 ± 104	285 ± 142	0.001
Saturated fat (%)^∗^	9.8 ± 5.6	10.4 ± 5.8	9.2 ± 5.4	0.121	N/A	9.8 ± 5.6	
Sugar (%)	12 ± 6	11 ± 6	13 ± 6	0.004	15 ± 6	11 ± 5	<0.001
Sugar (g)	43 ± 24	43 ± 25	43 ± 23	0.879	47 ± 24	41 ± 24	0.035
Fiber (g)	9 ± 4	9 ± 4	9 ± 4	0.959	9 ± 5	9 ± 4	0.699
Fiber (g/1000 kcal)	6 ± 3	6 ± 3	7 ± 3	<0.001	7 ± 3	6 ± 2	0.035
Sodium (mg)	2933 ± 1309	3094 ± 1449	2789 ± 1155	0.044	2143 ± 773	3271 ± 1346	<0.001
Carbohydrate (%)	52 ± 8	51 ± 8	52 ± 8	0.007	56 ± 8	51 ± 8	<0.001
Fat (%)	31 ± 7	31 ± 7	31 ± 6	0.076	28 ± 6	31 ± 7	<0.001
Protein (%)	17 ± 3	18 ± 3	17 ± 3	0.360	16 ± 3	18 ± 3	0.003

^∗^Data available in 213/304 cases. N/A: not available.

**Table 2:** Comparison of nutrient intakes between patients with good glycemic control (A1C < 7.0%) and those with poor glycemic control (*N* = 299 cases).

	Total (*n* = 299)		*p* value
	A1C < 7% (*n* = 158)	A1C ≥ 7% (*n* = 141)	
Energy (kcal)	1397 ± 405	1470 ± 445	0.135
Carbohydrate (g)	180 ± 50	191 ± 60	0.077
Protein (g)	58 ± 21	62 ± 21	0.098
Fat (g)	48 ± 20	51 ± 21	0.297
Cholesterol (mg)	262 ± 127	281 ± 140	0.226
Saturated fat (%)^∗^	10 ± 6	10 ± 5	0.637
Sugar (%)	12 ± 6	12 ± 6	0.963
Sugar (g)	41 ± 20	45 ± 27	0.153
Fiber (g)	8 ± 4	9 ± 4	0.080
Fiber (g/1000 kcal)	6 ± 2	6 ± 3	0.339
Sodium (mg)	2955 ± 1380	2922 ± 1237	0.826
Carbohydrate (%)	52 ± 8	52 ± 8	0.896
Fat (%)	31 ± 7	30 ± 6	0.929
Protein (%)	17 ± 3	17 ± 3	0.295

^∗^Data available in 213/299 cases.

**Table 3:** Comparison of nutrient intakes between people with good glycemic control (A1C < 7.0%) and those with poor glycemic control in each site of patients.

	Ramathibodi (*n* = 87)		*p* value	Theptarin (*n* = 212)		*p* value
	A1C < 7% (*n* = 38)	A1C ≥ 7% (*n* = 49)		A1C < 7% (*n* = 120)	A1C ≥ 7% (*n* = 92)	
Energy (kcal)	1232 ± 313	1318 ± 367	0.249	1449 ± 418	1552 ± 463	0.093
Carbohydrate (g)	172 ± 45	182 ± 57	0.378	182 ± 52	196 ± 61	0.077
Protein (g)	48 ± 15	54 ± 15	0.108	62 ± 22	67 ± 22	0.067
Fat (g)	39 ± 14	42 ± 16	0.367	51 ± 20	55 ± 22	0.149
Cholesterol (mg)	227 ± 107	244 ± 101	0.435	273 ± 131	300 ± 154	0.171
Saturated fat (%)^∗^	N/A	N/A	N/A	10 ± 6	10 ± 5	0.637
Sugar (%)	16 ± 6	14 ± 6	0.220	11 ± 5	11 ± 5	0.852
Sugar (g)	48 ± 22	46 ± 24	0.596	39 ± 19	44 ± 28	0.090
Fiber (g)	8 ± 5	9 ± 4	0.333	9 ± 3	9 ± 4	0.122
Fiber (g/1000 kcal)	6 ± 3	7 ± 4	0.364	6 ± 2	6 ± 2	0.930
Sodium (mg)	2013 ± 819	2230 ± 709	0.187	3254 ± 1390	3290 ± 1302	0.847
Carbohydrate (%)	56 ± 7	55 ± 8	0.618	51 ± 8	51 ± 7	0.818
Fat (%)	28 ± 6	28 ± 7	0.893	31 ± 7	32 ± 6	0.720
Protein (%)	16 ± 3	16 ± 3	0.241	17 ± 3	18 ± 3	0.322

^∗^Data available in 212/299 cases. N/A: not available.

**Table 4:** Results of diabetes self-care behavior based on validated DSCB questionnaire (*N* = 213 cases).

Dietary self-care behavior components	Advised to follow behavior during diabetes education sessions (%)	Never	Seldom	Sometimes	Often	Always
(1) Followed diabetes meal plan	70%	5%	14%	36%	32%	10%
(2) Had meals at approximately the same time and amount daily	68%	6%	13%	22%	31%	22%
(3) Followed diabetes exchange list	61%	13%	17%	31%	28%	9%
(4) Counted carbohydrate	38%	25%	23%	23%	20%	6%
(5) Reduced fat consumption	65%	5%	22%	36%	22%	12%
(6) Increased fiber intake	61%	5%	15%	28%	33%	15%
(7) Kept a food record daily	16%	56%	24%	6%	12%	0%

**Table 5:** Comparison of diabetes nutritional knowledge scores in patients with low and high adherence to dietary self-care behavior (*N* = 213 cases).

Dietary self-care behavior components	Scores of high adherence patients	Scores of low adherence patients	*p* value
(1) Followed diabetes meal plan	2.7 ± 1.2	2.6 ± 1.2	0.635
(2) Had meals at approximately the same time and amount daily	2.6 ± 1.2	2.8 ± 1.1	0.631
(3) Followed diabetes exchange list	2.7 ± 1.2	2.8 ± 1.1	0.631
(4) Counted carbohydrate	2.8 ± 1.2	2.7 ± 1.2	0.678
(5) Reduced fat consumption	2.7 ± 1.2	2.7 ± 1.1	0.918
(6) Increased fiber intake	2.7 ± 1.2	2.9 ± 1.0	0.330
(7) Kept a food record daily	2.3 ± 1.2	2.6 ± 1.1	0.582

**Table 6:** Comparison of dietary intake in patients with low and high adherence to dietary self-care behavior.

	Adherence level	Energy (kcal)	Protein (g)	CHO (g)	Sugar (g)	Fat (g)	SFA (g)	Cholesterol (mg)	Fiber (g)	Sodium (mg)
(1) Followed diabetes meal plan	High	1516 ± 401	66 ± 22	189 ± 56	40 ± 24	55±19	16±7	291 ± 141	9.2 ± 3.7	3309 ± 1373
	Low	1558 ± 544	66 ± 25	198 ± 63	44 ± 27	56 ± 27	16 ± 9	291 ± 142	8.9 ± 4.3	3504 ± 1454
(2) Had meals at approximately the same time and amount daily	High	1555 ± 412	67 ± 22	193 ± 57	42 ± 25	57 ± 20	16 ± 7	292 ± 136	9.4 ± 3.9	3382 ± 1359
	Low	1430 ± 524	64 ± 27	182 ± 56	40 ± 22	49 ± 26	14 ± 9	299 ± 170	8.6 ± 3.4	3397 ± 1527
(3) Followed diabetes exchange list	High	1552 ± 426	69 ± 24^∗^	194 ± 58	41 ± 23	56 ± 20	16 ± 7	302 ± 153	9.5 ± 4.0^∗^	3542 ± 1576^∗^
	Low	1425 ± 439	59 ± 19	184 ± 59	38 ± 23	50 ± 21	15 ± 8	255 ± 114	8.3 ± 2.8	3054 ± 1037
(4) Counted carbohydrate	High	1591 ± 459	70 ± 26	205 ± 66	40 ± 25	54 ± 20	15 ± 6	304 ± 150	9.5 ± 3.9	3467 ± 1450
	Low	1439 ± 442	62 ± 21	180 ± 54	40 ± 21	52 ± 22	14 ± 8	264 ± 129	9.0 ± 2.9	3378 ± 1274
(5) Reduced fat consumption	High	1523 ± 428	66 ± 23	189 ± 56	42 ± 26	56 ± 22	16 ± 8	290 ± 145	9.3 ± 3.7	3353 ± 1439
	Low	1517 ± 497	66 ± 22	188 ± 58	38 ± 19	55 ± 22	14 ± 7	296 ± 140	8.9 ± 3.5	3298 ± 1259
(6) Increased fiber intake	High	1494 ± 413	65 ± 22	188 ± 56	42 ± 26	54 ± 20	15 ± 7	291 ± 146	9.1 ± 3.7	3284 ± 1367
	Low	1470 ± 454	66 ± 24	181 ± 50	35 ± 14	54 ± 23	12 ± 7	279 ± 130	9.1 ± 3.4	3368 ± 1334
(7) Kept a food record daily	High	1451 ± 259	56 ± 14	199 ± 49	31 ± 15	48 ± 15	14 ± 10	220 ± 93	9.3 ± 5.0	3655 ± 1398
	Low	1498 ± 409	64 ± 21	189 ± 55	43 ± 27	54 ± 21	15±8	235 ± 106	10.4 ± 3.8	3101 ± 1248

^∗^Statistically significant differences between low and high adherence levels in each nutrient (*p* < 0.05).

**Table 7:** Results of multiple regression analysis for independent factors which predicted glycemic control.

	Beta coefficients		*p* value	95% confidence interval	
	Unstandardized	Standardized			
Insulin (yes/no)	1.003	0.330	**0.001**	0.662	1.344
DM duration (years)	0.024	0.172	**0.007**	0.007	0.041
Age (years)	−0.012	−0.105	0.097	−0.026	0.002
Sex (male or female)	−0.045	−0.018	0.752	−0.322	0.233
Daily energy intakes (kcal)	−0.001	−0.501	0.258	−0.004	0.001
Carbohydrate intakes (gram)	0.007	0.330	0.186	−0.004	0.018
Free sugar intake (g)	−0.001	−0.012	0.866	−0.008	0.007
Protein intakes (g)	0.008	0.130	0.299	−0.007	0.022
Fat intakes (g)	0.011	0.172	0.407	−0.014	0.035
BMI (kg/m^2^)	0.018	0.070	0.218	−0.011	0.048

**Table 8:** Comparison of nutrient intakes from our patients with the latest ADA recommendation in 2014.

Topics	MNT recommendations from ADA (MNT 2014)	Clinical practice guideline for diabetes in Thailand 2017	Our data
Energy (kcal/d)	Adjust based on age, weight, and height	Adjust based on age, weight, and height	1427 ± 425
Carbohydrate (% of total kcal/d)	No ideal percentage^∗^	No ideal percentage^∗^	52%
Sugar (% of total kcal)	—	≤5% (3–6 teaspoons)	12 ± 6%
Fiber (g/d)	>25	>25	9 ± 4
Fiber (g/1000 kcal/d)	≥14	≥14	6 ± 3
Protein (% of total kcal/d)	No ideal percentage^∗^	15–20% in people without diabetic kidney disease	17%
Total fat (% of total kcal/d)	No ideal percentage^∗^	No ideal percentage^∗^	31%
Saturated fatty acids (% of total kcal/d)	<10% or <7% in people with dyslipidemia	≤7%	10 ± 6%
Total cholesterol (mg/d)	<300 mg/d or <200 mg/d in people with dyslipidemia	—	270 ± 134
Sodium (mg/d)	<2300	<2000	2933 ± 1309

^∗^No ideal percentage: the guideline suggests that there is not an ideal percentage of calories from carbohydrate, protein, and fat for all people with diabetes; the Thai guideline in 2017 also adopted the ADA recommendation.

## Data Availability

The data used to support the findings of this study are available from the corresponding author upon request.

## Supplementary Materials

Supplementary Materials Appendix 1. The detailed of adapted “Theptarin DM questionnaire” which assesses individuals' knowledge of diabetes nutritional knowledge. The questionnaire consists of 5 questions and the scores were classified into three categories: low (score less than 3), moderate (score 3-4), and high (score = 5). Appendix 2. The details of “dietary self-care behavior (DSCB)” which assess patients' reports of the self-care recommendations they had received from their healthcare providers and adherence to seven reported diet-related self-care behaviors. Appendix 3. Demographic and clinical characteristics of patients (*N* = 304). Appendix 4. Comparison of demographic data between patients with good glycemic control (A1C < 7.0%) and those with poor glycemic control (*N* = 299 cases). Appendix 5. Adjusted daily energy intakes and nutrient intakes after exclusion of underreporters.
