# Supplementary material for: Evaluation of Dietary Intakes and Nutritional Knowledge in Thai Patients with Type 2 Diabetes Mellitus
Source: J Diabetes Res. 2018 Dec 20;2018:9152910. doi: 10.1155/2018/9152910 (PMC6317123; doi:10.1155/2018/9152910)
Supplement: Supplementary Materials — Appendix 1. The detailed of adapted “Theptarin DM questionnaire” which assesses individuals' knowledge of diabetes nutritional knowledge. The questionnaire consists of 5 questions and the scores were classified into three categories: low (score less than 3), moderate (score 3-4), and high (score = 5). Appendix 2. The details of “dietary self-care behavior (DSCB)” which assess patients' reports of the self-care recommendations they had received from their healthcare providers and adherence to seven reported diet-related self-care behaviors. Appendix 3. Demographic and clinical characteristics of patients (N = 304). Appendix 4. Comparison of demographic data between patients with good glycemic control (A1C < 7.0%) and those with poor glycemic control (N = 299 cases). Appendix 5. Adjusted daily energy intakes and nutrient intakes after exclusion of underreporters. [file 9152910.f1.docx]

**Supplementary Materials**

**Appendix 1** The detailed of **adapted ‘THEPTARIN DM questionnaire’** which assesses individuals’ knowledge of diabetic nutrition knowledge. The questionnaire consists of 5 questions and were classified into three categories: low (less than 3), moderate (score 3-4), and high (score = 5)

**
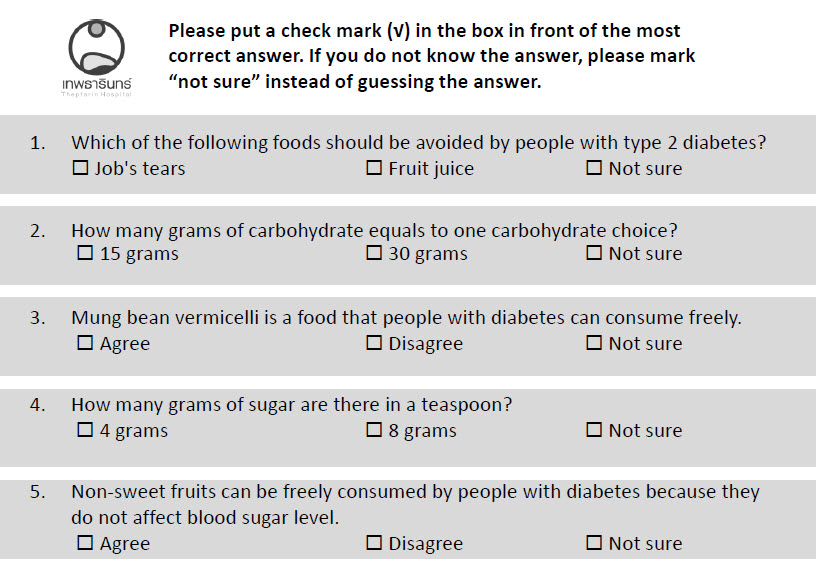
**

**Appendix 2** The detailed of **‘Dietary self-care behavior (DSCB)**’ to assess patients’ reports of the self-care recommendations they had received from their health care providers and adherence to seven reported diet related self-care behaviors


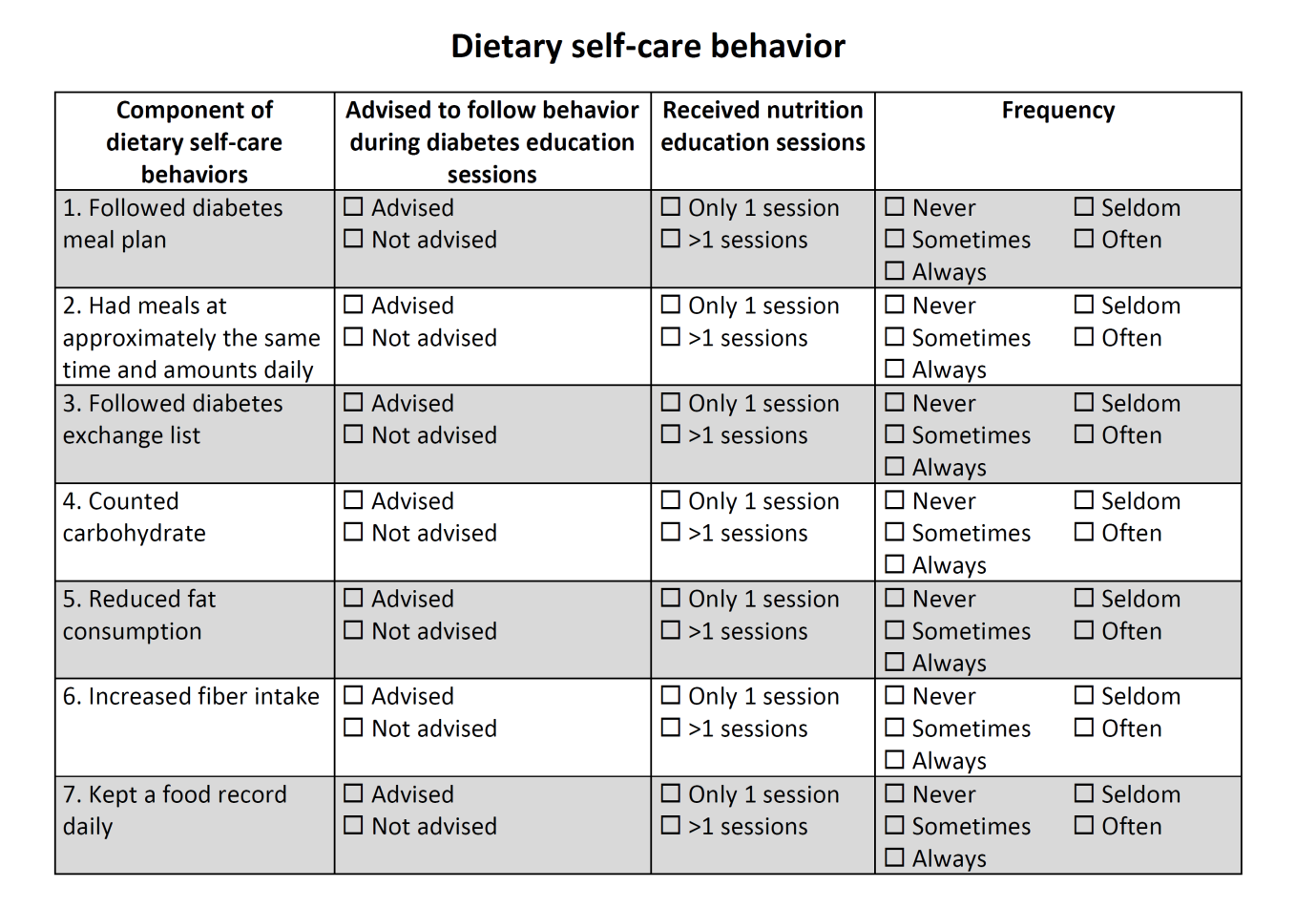


**Reference:** Ouyang CM, Dwyer JT, Jacques PF, Chuang LM, Haas CF, Weinger K. Determinants of dietary self-care behaviors among Taiwanese patients with type 2 diabetes. Asia Pac J Clin Nutr. 2015;24:430-7.

**Appendix 3.** Demographic and clinical characteristics of patients (N=304)

|  | Total participants  (n=304) | Total (n=304) | |  | Total (n=304) | |  |
| --- | --- | --- | --- | --- | --- | --- | --- |
|  |  | **Men**  **(n=144)** | **Women**  **(n=160)** | ***p-value*** | **Ramathibodi**  **(n=91)** | **Theptarin**  **(n=304)** | ***p-value*** |
| Age (years) | 57.4±10.9 | 57.4±10.5 | 57.4±11.3 | 0.974 | 55.1±11.5 | 58.4±10.5 | 0.014 |
| Height (cm) | 162±10 | 170±7 | 155±6 | <0.001 | 160±9 | 163±10 | 0.018 |
| Weight (kg) | 72±15 | 78±15 | 67±14 | <0.001 | 73±15 | 72±15 | 0.398 |
| BMI (kg/m^2^) | 27.3±4.8 | 27.1±4.5 | 27.5±5.1 | 0.529 | 28.5±5.2 | 26.8±4.5 | 0.005 |
| <18.5 | 3 (1%) | 2 (1%) | 1 (1%) |  | 0 (0%) | 3 (1%) |  |
| 18.5-22.9 | 51 (17%) | 19 (13%) | 32 (20%) |  | 12 (13%) | 39 (18%) |  |
| 23.0-24.9 | 50 (16%) | 29 (20%) | 21 (13%) |  | 15 (16%) | 35 (16%) |  |
| ≥25.0 | 200 (66%) | 94 (65%) | 106 (66%) |  | 64 (70%) | 136 (64%) |  |
| DM Duration (years) | 11.7±9.0 | 13.0±8.5 | 10.6±9.15 | 0.020 | 10.9±9.2 | 12.1±8.9 | 0.319 |
| A1C (%NGSP) | 7.2±1.2 | 7.3±1.4 | 7.1±1.6 | 0.214 | 7.5±1.4 | 7.1±1.1 | 0.004 |
| Cholesterol (mg/dl)* | 164±36 | 161±38 | 167±34 | 0.407 | N/A | 164±36 |  |
| LDL-C (mg/dl)* | 96±35 | 100±41 | 93±27 | 0.111 | N/A | 96±35 |  |
| HDL-C (mg/dl)* | 56±19 | 52±14 | 60±22 | 0.004 | N/A | 56±19 |  |
| Triglyceride (mg/dl)* | 140±90 | 142±90 | 139±89 | 0.082 | N/A | 140±90 |  |
| Creatinine (mg/dl)* | 0.8±0.2 | 0.9±0.2 | 0.7±0.1 | 0.214 | N/A | 0.8±0.2 |  |
| eGFR (ml/min/1.73 m^2^) | 92±20 | 90±26 | 93±24 | 0.147 | 88±26 | 93±17 | 0.125 |
| Systolic BP (mmHg) | 128±17 | 129±19 | 127±24 | 0.136 | 126±14 | 129±17 | 0.005 |
| Diastolic BP (mmHg) | 73±10 | 73±12 | 72±14 | 0.501 | 75±8 | 71±11 | 0.001 |
| Hypertension (%) | 180(60.0%) | 87(60.4%) | 93(58.1%) | 0.777 | 63 (69%) | 117 (55%) | 0.005 |
| Dyslipidemia (%) | 263(87.7%) | 124(86.1%) | 139(86.9%) | 0.632 | 82 (90%) | 181 (85%) | 0.027 |
| Current smoker (%)* | 18(8.5%) | 16(14.9%) | 2(1.9%) |  | N/A | 18 (8%) |  |
| Pattern of diabetes treatment |  |  |  |  |  |  |  |
| Diet control alone (%) | 14(4.6%) | 5(3.5%) | 9(5.6%) | 0.371 | 2 (2%) | 12 (4%) | 0.191 |
| Metformin (%) | 252(82.9%) | 119(82.6%) | 133(83.1%) | 0.911 | 77 (85%) | 175 (58%) | 0.603 |
| Sulfonylurea (%) | 116(38.2%) | 54(37.5%) | 62(38.8%) | 0.823 | 46 (51%) | 70 (23%) | 0.004 |
| DPP4 inhibitor (%) | 128(42.1%) | 62(43.1%) | 66(41.3%) | 0.750 | 28 (31%) | 100 (33%) | 0.009 |
| SGLT2 inhibitor (%) | 27(8.9%) | 15(10.4%) | 12(7.5%) | 0.589 | 0 (0%) | 27 (9%) |  |
| Thiazolidinediones (%) | 110(36.2%) | 68(47.2%) | 42(26.3%) | <0.001 | 21 (23%) | 89 (29%) | 0.002 |
| α-glucosidase inhibitor (%) | 6(2.0%) | 2(1.4%) | 4(2.5%) | 0.487 | 5 (5%) | 1 (0%) | 0.004 |
| GLP1 agonist (%) | 6(2.0%) | 3(2.1%) | 3(1.9%) | 0.896 | 4 (4%) | 2 (1%) | 0.047 |
| Insulin usage (%) | 63(20.7%) | 36(25.0%) | 27(16.9%) | 0.081 | 27 (30%) | 36 (12%) | 0.012 |
| Education^#^ |  |  |  | 0.149 |  |  | 0.557 |
| Primary | 59(21%) | 21(16%) | 38(26%) |  | 15 (18%) | 44 (23%) |  |
| Secondary | 52(19%) | 23(18%) | 29(20%) |  | 13 (16%) | 40 (21%) |  |
| University | 165(60%) | 86(66%) | 79(54%) |  | 54 (66%) | 111 (56%) |  |

*Data available in 213/304 cases

^#^Data available in 276/304 cases

N/A – Not Available

**Appendix 4.**Comparison of demographic data between patients with good glycemic control (A1C<7.0%) and those with poor glycemic control (N=299 cases)

|  | A1C<7%  (n=158) | A1C≥7%  (n=141) | p-value |
| --- | --- | --- | --- |
| Age (years) | 58.0±10.3 | 56.9±11.6 | 0.398 |
| Female (%) | 90 (57%) | 66 (47%) | 0.079 |
| BMI (kg/m^2^) | 26.8±4.6 | 27.7±4.7 | 0.130 |
| DM Duration (years) | 10.1±8.3 | 13.6±9.4 | 0.001 |
| Pattern of diabetes treatment |  |  |  |
| Diet control alone (%) | 13 (8%) | 1 (1%) | 0.002 |
| Metformin (%) | 127 (80%) | 120 (85%) | 0.282 |
| Sulphonylurea (%) | 45 (28%) | 70 (50%) | 0.000 |
| DPP4 inhibitor (%) | 57 (36%) | 68 (48%) | 0.033 |
| SGLT2 inhibitor (%) | 6 (5%) | 21 (23%) | 0.000 |
| Thiazolidinedione (%) | 57 (36%) | 53 (38%) | 0.787 |
| α-glucosidase inhibitor (%) | 1 (1%) | 5 (4%) | 0.073 |
| GLP1 agonist (%) | 2 (1%) | 4 (3%) | 0.334 |
| Insulin usage (%) | 14 (9%) | 48 (34%) | 0.000 |
| Educational level |  |  | 0.810 |
| Primary | 31 (20%) | 27 (19%) |  |
| Secondary | 27 (17%) | 25 (18%) |  |
| University | 83 (53%) | 79 (56%) |  |

**Appendix 5.** Adjusted daily energy intakes and nutrients intakes after exclusion of under-reporters

| **Under-reporting detection methods** | **Static cut-off^1^**  **(500-5,000 Kcal)** | **Goldberg *et al*.^2^** | **Henry *et al*.^3^** | **Mifflin *et al*.^4^** |
| --- | --- | --- | --- | --- |
| n (%) | 304 (100%) | 65 (21%) | 88 (29%) | 86 (28%) |
| Energy intake (Kcal) | 1,427±425 | 1,932±401 | 1,683±477 | 1,848±390 |
| min – max (Kcal) | 641-2,743 | 1,243-2,743 | 1,032-2,743 | 1,139-2,743 |
| Carbohydrate (g) | 185±55 | 239±50 | 212±57 | 231±49 |
| Protein (g) | 60±21 | 79±24 | 70±24 | 76±23 |
| Fat (g) | 49±20 | 71±22 | 60±23 | 67±22 |
| Sugar (g) | 43±24 | 58±28 | 51±25 | 57±28 |
| Fiber (g) | 9±4 | 12±5 | 11±4 | 11±4 |
| Sodium (mg) | 2,933±1,309 | 3,829±1,529 | 3,382±1,506 | 3,661±1,457 |
| %Carbohydrate | 52 | 50 | 50 | 50 |
| %Protein | 17 | 16 | 17 | 17 |
| %Fat | 31 | 33 | 33 | 33 |

**References**

1. Willett W. Nutritional Epidemiology. Oxford: Oxford University Press; 2012.
2. Goldberg GR, Black AE, Jebb SA, Cole TJ, Murgatroyd PR, Coward WA, et al. Critical evaluation of energy intake data using fundamental principles of energy physiology: Derivation of cut-off limits to identify under-reporting. Eur J Clin Nutr. 1991;45: 569-81.
3. Henry CJ. Basal metabolic rate studies in humans: measurement and development of new equations. Public Health Nutr. 2005;8:1133-52.
4. Mifflin MD, St. Jeor ST, Hill LA, Scott BJ, Daugherty SA, Koh YO. A new predictive equation for resting energy expenditure in healthy individuals. Am J Clin Nutr. 1990;51:241-7.
